# Penicillin-resistant *Streptococcus dysgalactiae* subsp. *equisimilis* and the association with penicillin-binding protein mutations: a population genotypic and phenotypic study

**DOI:** 10.1128/aac.00137-26

**Published:** 2026-08-03

**Authors:** Ouli Xie, Aimee Tan, Thanh Binh Nguyen, Andrew J. Hayes, Ravindra Dotel, Glen Carter, Matthew V. N. O'Sullivan, Katherine Bond, Christopher A. McDevitt, Tony M. Korman, Bart J. Currie, David B. Ascher, Steven Y. C. Tong, Sebastiaan J. van Hal, Mark R. Davies

**Affiliations:** 1 Department of Microbiology and Immunology, University of Melbourne, The Peter Doherty Institute for Infection and Immunity, Melbourne, Victoria, Australia; 2 Department of Infectious Diseases, University of Melbourne, The Peter Doherty Institute for Infection and Immunity, Melbourne, Victoria, Australia; 3 Monash Infectious Diseases, Monash Health, Melbourne, Victoria, Australia; 4 Global and Tropical Health Division, Menzies School of Health Research, Charles Darwin University 10095, Darwin, Northern Territory, Australia; 5 School of Chemistry and Molecular Biosciences, University of Queensland 198110, St Lucia, Queensland, Australia; 6 Department of Infectious Diseases, Blacktown Hospital, Sydney, NSW, Australia; 7 Doherty Applied Microbial Genomics, Department of Microbiology and Immunology, The University of Melbourne at the Peter Doherty Institute for Infection and Immunity, Melbourne, Victoria, Australia; 8 Department of Infectious Diseases, Westmead Hospital, Sydney, NSW, Australia; 9 Faculty of Medicine and Health, The University of Sydney 4334, Sydney, NSW, Australia; 10 NSW Health Pathology, Westmead Hospital, Sydney, NSW, Australia; 11 Victorian Infectious Diseases Service, The Royal Melbourne Hospital, The Peter Doherty Institute for Infection and Immunity, Melbourne, Victoria, Australia; 12 Department of Microbiology, The Royal Melbourne Hospital 90134, Melbourne, Victoria, Australia; 13 Centre for Inflammatory Diseases, Monash University 2541, Melbourne, Victoria, Australia; 14 Infectious Diseases Department, Royal Darwin Hospital, Darwin, Northern Territory, Australia; 15 Faculty of Medicine and Health, Central Clinical School, The University of Sydney 4334, Sydney, NSW, Australia; 16 Department of Infectious Diseases and Microbiology, Royal Prince Alfred Hospital, Sydney, NSW, Australia; Shionogi Inc., Florham Park, New Jersey, USA

**Keywords:** bacterial population genomics, *Streptococcus pyogenes*, *Streptococcus dysgalactiae*, penicillin resistance, antimicrobial resistance, *Streptococcus*

## Abstract

*Streptococcus dysgalactiae* subspecies *equisimilis* (SDSE) is increasingly recognized as a cause of human disease. Despite decades of penicillin use, SDSE is near universally penicillin-susceptible. Here we report and characterize by whole genome sequencing (WGS) three penicillin-resistant SDSE isolates (minimum inhibitory concentration [MIC] ≥0.256 mg/L) isolated from blood and skin in an individual with recurrent cellulitis on penicillin prophylaxis. The isolates carried 11 penicillin-binding protein (PBP) PBP2x, five PBP1a, and one PBP2b mutations that were distinct from the only previously reported WGS-characterized penicillin-resistant SDSE isolates (*n* = 4). We then took a genotype-led approach to test the MICs of penicillin and amoxicillin for 63 isolates with PBP2x, PBP1a, and PBP2b variants selected from a well-characterized database of invasive SDSE isolates from Australia (2011–2023, *n* = 436). PBPs were highly conserved in Australian invasive SDSE genomes. Only a single isolate carrying a PBP2x variant with five mutations around the penicillin-binding cleft found in 1/436 invasive isolates demonstrated a four-fold rise in penicillin MIC, although remaining below clinical breakpoints. Strains with PBP2b and PBP1a variants alone were not associated with raised MICs. To provide broader context for PBP variants, we then examined a global database of SDSE genomes (*n* = 1,160). PBP2x mutations in isolates with raised and/or resistant penicillin MICs were found in only nine global SDSE database genomes. SDSE penicillin resistance is rare and associated with multiple PBP mutations, particularly PBP2x. WGS with phenotypic testing of novel PBP variants could be used to monitor emerging mutations associated with raised penicillin MICs.

## INTRODUCTION

*Streptococcus dysgalactiae* subspecies *equisimilis* (SDSE, commonly referred to as Group C/G *Streptococcus*) is increasingly recognized as a cause of serious human disease (1). SDSE is closely related to *Streptococcus pyogenes* with overlapping disease manifestations (1, 2). Incidence of invasive SDSE disease is comparable or even exceeds that of *S. pyogenes* in some countries, but surveillance lags behind that of its more well-studied relative (2–4).

Despite decades of penicillin use, SDSE remains nearly universally penicillin susceptible (5). Emergent lineages of *S. pyogenes* associated with raised minimum inhibitory concentrations (MICs) to beta-lactams, albeit lower than clinical breakpoints, have been described (6, 7). To our knowledge, only four penicillin-resistant, i.e., above clinical breakpoints, SDSE isolates from three individuals have been reported and characterized by whole genome sequencing (WGS) (8). Increased penicillin MICs in other streptococci, notably *Streptococcus pneumoniae,* are mediated by mutations in penicillin-binding proteins (PBPs), of which the transpeptidase domain of PBP2x is considered critical (9). Automated antimicrobial phenotypic testing would be unlikely to detect emergent lineages with increased penicillin MICs below the breakpoint as many are not designed to interpret subtle changes in MIC below breakpoints. Large-scale WGS-based surveillance for reduced penicillin susceptibility has been performed for *S. pyogenes*, *S. agalactiae,* and *S. pneumoniae* but not for SDSE (9–11).

We report a case of penicillin resistance in an individual with invasive SDSE disease characterized by WGS with multiple PBP mutations. To examine the circulating diversity of PBP variants, we leveraged a previously published data set of 436 invasive SDSE isolates from Australia to undertake a genotype-informed analysis by testing penicillin MICs of isolates with unique PBP genotypes. Finally, we contextualized our analysis by examining the prevalence of mutations found in isolates with raised penicillin MICs or penicillin resistance in a global SDSE genome database encompassing 1,160 genomes from 19 different countries.

## MATERIALS AND METHODS

### Resistant *Streptococcus dysgalactiae* subsp. *equisimilis* isolates

Three isolates of *Streptococcus dysgalactiae* collected from routine clinical care in 2015 from a single patient (blood *n* = 2, skin swab *n* = 1), putatively identified by MALDI-TOF MS (Bruker MALDI Biotyper, Bruker Daltoniks, Billerica, MA using Bruker server version 4.1.100 [PYTH]), were found to be penicillin-resistant by disc diffusion using CLSI methodology (susceptible ≥24 mm) (12). These isolates were subsequently confirmed to be SDSE by WGS read classification using Kraken2 v2.1.2 (13).

### Molecular analyses

For the penicillin-resistant isolates, single bacterial colonies were selected for DNA extraction using QIAsymphony DSP Virus/Pathogen Mini Kit (Qiagen, Hilden, Germany) and libraries prepared using Illumina Nextera XT DNA Library Preparation Kits (Illumina, San Diego, USA). Whole genome sequencing was performed using the Illumina NextSeq 550 platform (San Diego, USA) to generate 150-bp paired-end reads.

Genomes were assembled using previously described pipelines (14, 15). Sequences for *pbp2x*, *pbp2b,* and *pbp1a,* which have been shown to be critical for mediating penicillin resistance or raised MICs in *Streptococcus pneumoniae, Streptococcus agalactiae,* and *S. pyogenes,* were extracted from genome assemblies using screen_assembly v1.2.7 with reference sequences from GGS_124 (NC_012891.1) and *in silico* translated to amino acid sequences (9, 16–18). PBP mutations were compared to three previously described penicillin-resistant SDSE cases (8). Active-site motifs in PBP2x were determined from homology with *S. pyogenes* and included 340–343 STMK, 399–401 SSN, 550–552 KSG (19).

Molecular epidemiological markers, *emm*-type, and MLST were inferred using emmtyper v0.2.0 (https://github.com/MDU-PHL/emmtyper) and MLST v2.23.0 (https://github.com/tseemann/mlst) (20). Global genomic sequence clusters (highly concordant with lineage) were assigned by PopPUNK v2.8.0 using version 2 of the global SDSE model (https://bacpop.org/poppunk-databases/) (2). *In silico* search for beta-lactamases was performed using Abritamr v1.0.14 at default settings (21).

### Data sets to provide broader context of PBP mutations in SDSE

To provide a broader context, the phenotypically tested isolates were contextualized within two published, diverse whole-genome data sets. i) Invasive SDSE in geographically diverse regions of Australia (*n* = 436 genomes) collected retrospectively from five hospital networks across temperate urbanized Melbourne and Sydney and the tropical Top End of the Northern Territory, 2011–2023 (2). These previously described isolates with corresponding WGS were obtained from sterile sites during routine clinical care and were selected as they were generally representative of globally circulating lineages and available for antimicrobial susceptibility testing (Table S1a; 2). ii) A global database of high-quality SDSE genomes isolated from humans encompassing 19 countries in seven different regions (*n* = 1,160 genomes) collated from publicly available genomes as of 15 January 2024 (2, 14). From the original global database, five genomes had frameshifted *pbp2x* genes likely due to genome assembly errors and one had an incompletely assembled *pbp2x* gene and were excluded. All isolates were confirmed to be SDSE by WGS-level read classification as above.

### Association between circulating PBP variants in Australia and penicillin MIC

PBP2x, PBP2b, and PBP1a amino acid mutations were inferred from WGS data from Australian invasive isolates. Wild-type (WT) profiles of PBP2x, PBP2b, and PBP1a were defined as the most prevalent amino acid sequences in Australian invasive SDSE isolates as these were previously found to be representative of global lineages and were available for susceptibility testing (2). Frequency of amino acid mutations in PBP2x was mapped onto the predicted AlphaFold2 structure of PBP2x (AF-C5WIJ2-F1) using ChimeraX v1.6.1 (22).

### Broth microdilution susceptibility testing

Given the importance of PBP2x, PBP2b, and PBP1a in determining penicillin susceptibility in other streptococci, we selected a genome-informed subset of 63/436 Australian invasive SDSE isolates representative of strains with unique PBP2x, PBP2b, and PBP1a amino acid profiles and the three penicillin-resistant isolates in this study for penicillin and amoxicillin susceptibility testing by broth microdilution. Briefly, isolates were cultured on horse blood agar overnight aerobically at 37°C. Amoxicillin or penicillin were two-fold serially diluted in cation adjusted Mueller Hinton broth and dilutions dispensed across 96-well flat-bottomed microtitre plates. Strain inocula were prepared by direct suspension of colonies from agar plates, dispensed at 2–8 × 10^4^ CFU per well in the antibiotic plates and incubated at 37 °C. At 20 h, the MIC was determined as the lowest antibiotic concentration without visible growth. All isolates were tested by the same blinded operator in triplicate. Penicillin resistance was defined as MIC >0.03 mg/L in accordance with EUCAST clinical breakpoints (v15.0) (5). Amoxicillin MIC was determined by the same method and resistance interpretation was extrapolated from penicillin as per EUCAST guidance (5). A significantly raised penicillin MIC was defined as a MIC four-fold higher (≥0.016 mg/L) than the modal MIC of strains with the WT PBP profile but below the clinical breakpoint.

### Modelling PBP2x-penicillin complex using docking and molecular dynamic simulations

To infer the potential mechanism leading to raised penicillin MIC, the PBP2x-penicillin complex was first generated by docking of penicillin to the AlphaFold 2 structure of PBP2x (AF-C5WIJ2-F1) using Autodock Vina v1.2.3 (23). Molecular interactions between PBP2x and penicillin were then analyzed using Arpeggio (24). To further infer the effects of mutations not predicted to be involved in direct binding interactions to penicillin, molecular dynamics (MD) simulations were performed using GROMACS (v2022.3) (Supplementary Methods) (25).

### Global prevalence of PBP2x variants associated with raised penicillin MICs

To examine the broader distribution of PBP mutations associated with raised penicillin MICs, PBP2x, PBP2b, and PBP1a variants were extracted from a global database of 1,160 SDSE genomes collated from publicly available genomes (2, 14). These were combined in a phylogeny with all available data sets (total *n* = 1,603 genomes), including (i) the three penicillin-resistant isolates from this study, (ii) 436 invasive SDSE isolates from Australia (2) (including the 63 that had antimicrobial susceptibility testing undertaken), and (iii) four penicillin-resistant isolates reported by Fuursted et al. (8). A core genome (sites present in at least 95% of genomes) single nucleotide polymorphism (SNP) alignment was generated using Snippy v4.6.0 (https://github.com/tseemann/snippy) against reference genome GGS_124 (NC_012891.1) with mobile genetic element regions masked. A maximum likelihood phylogeny was inferred using IQ-tree (v2.2.2.7) under a GTR + F + G4 model with 1,000 ultrafast nonparametric bootstrap replicates (26, 27).

## RESULTS

### Case report and identification of penicillin-resistant SDSE

A 65-year-old male presented with cellulitis on a background of diabetes. The patient had been on penicillin prophylaxis for 3 years for recurrent cellulitis with lower limb ulcers. Blood culture bottles (*n* = 2) taken in the emergency department flagged positive for *S. dysgalactiae* by MALDI-TOF species identification, which was subsequently confirmed to be SDSE by WGS. A superficial skin swab collected 10 days later from a lower limb wound also isolated SDSE.

**Fig 1:**
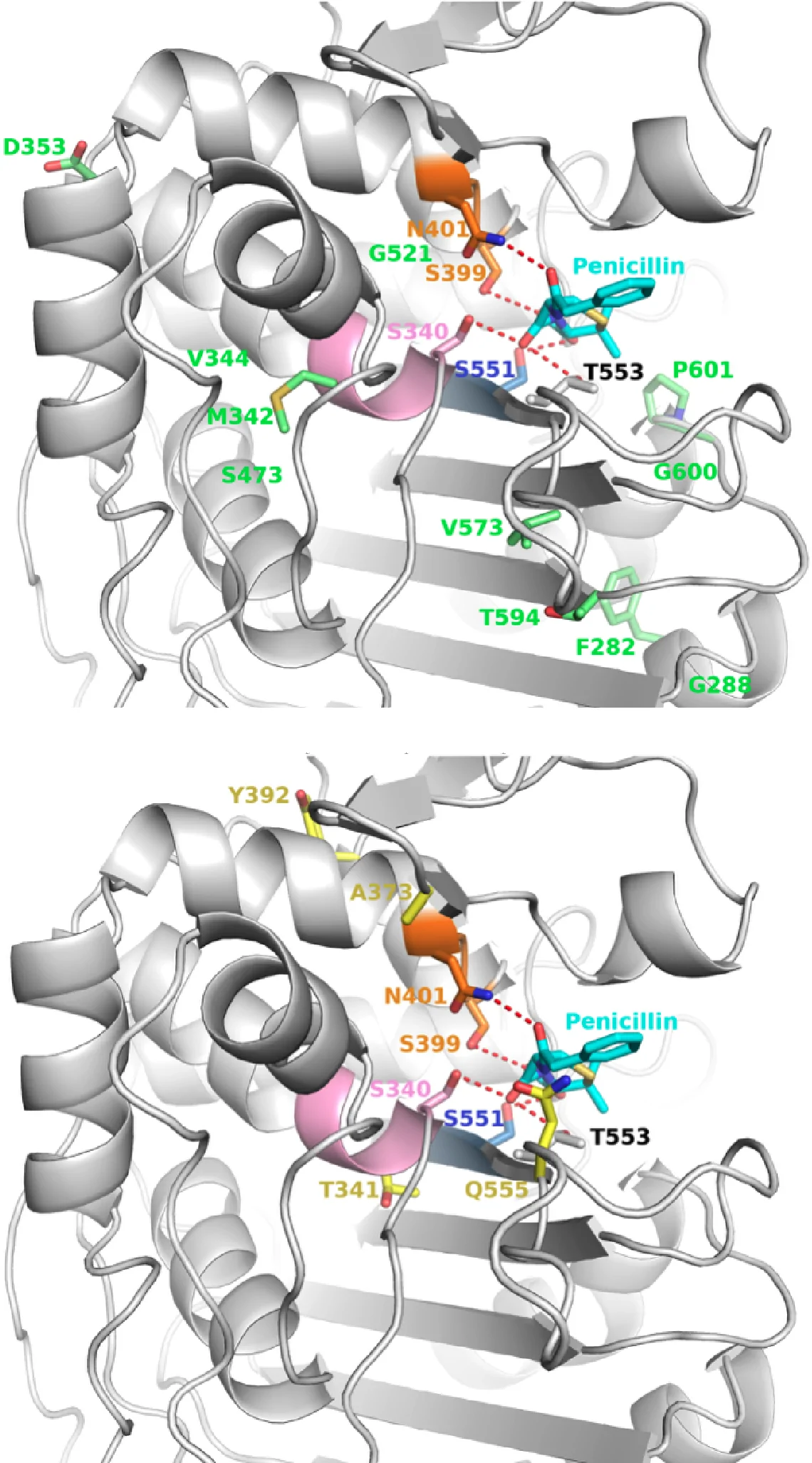
Amino acid mutations of penicillin-resistant *Streptococcus dysgalactiae* subsp. *equisimilis* isolates mapped against the predicted structure of PBP2x (AlphaFold structure AF-C5WIJ2-F1) with penicillin docked. The view is focused on the penicillin-binding pocket. The STMK, SSN, and KSG active-site motifs are highlighted in pink, orange, and blue, respectively. The position of mutated amino acids from penicillin-resistant isolates in this study is highlighted in the top panel in green (F282, G288, M342, V344, D353, S473, G521, V573, T594, G600, and P601). The position of mutated amino acids from previously reported penicillin-resistant isolates by Fuursted et al. is highlighted in the bottom panel in yellow (T341, A373, Y392, and Q555) (8). Hydrogen-bond interactions with penicillin are shown in red dashed lines, with nitrogen and oxygen atoms highlighted in blue and red.

All three isolates were closely related and initially tested penicillin-resistant by disc diffusion susceptibility testing, which was confirmed by broth microdilution (MIC 0.256–0.512 mg/L). The skin isolate (91570) differed from blood culture isolates by 32-35 SNPs indicative of within-host variation (2). All isolates harbored the same 11 novel amino acid substitutions in PBP2x, including M342I within and M344L adjacent to the STMK active site motif of PBP2x (Table 1; Fig. 1). They also harbored the same single PBP2b mutation and five PBP1a substitutions (Table 1). No beta-lactamase genes were detected.

**TABLE 1:** Antimicrobial susceptibility and penicillin-binding protein mutations in three penicillin-resistant *Streptococcus dysgalactiae* subsp. *equisimilis* isolates from this study and four previously reported isolates by Fuursted et al. (8) ^*b*^

Isolate	Site	Study	Penicillin MIC (mg/L)	Amoxicillin MIC^*a*^ (mg/L)	*emm*-type	MLST	Global genomic sequence cluster	PBP2x mutations	PBP2b mutations	PBP1a mutations
91512	Blood	Current	0.256 (R)	1.024	*stC*6979.0	9	5	F282V, G288C, M342I, V344L, D353E, S473P, G521E, V573I, T594K, G600D, P601L	A583T	I234V, P405S, T546I, A625T, D724E
91570	Skin	Current	0.512 (R)	1.024	*stC*6979.0	9	5	F282V, G288C, M342I, V344L, D353E, S473P, G521E, V573I, T594K, G600D, P601L	A583T	I234V, P405S, T546I, A625T, D724E
92158	Blood	Current	0.256 (R)	1.024	*stC*6979.0	9	5	F282V, G288C, M342I, V344L, D353E, S473P, G521E, V573I, T594K, G600D, P601L	A583T	I234V, P405S, T546I, A625T, D724E
1-2010	Blood	Fuursted	0.5 (R)	NR	*stG*5073.0	813	Novel	T341P, Y392N, Q555E	D223E	L411F, T587I, A589V, D724E
2-2010	Blood	Fuursted	0.5 (R)	NR	*stG*5073.0	813	Novel	T341P, A373V, Y392N, Q555E	D223E	L411F, T587I, A589V, D724E
2-2012	Blood	Fuursted	0.5 (R)	NR	*stG*5073.0	813	Novel	T341P, Y392N, Q555E	D223E	L411F, T587I, A589V, D724E
3-2012	Blood	Fuursted	0.5 (R)	NR	*stG*5073.0	813	Novel	T341P, A373V, Y392N, Q555E	D223E	L411F, T587I, A589V, D724E

^*a*^ Amoxicillin interpretation inferred from benzylpenicillin as per EUCAST Clinical Breakpoint Tables v15.0.

^*b*^ Minimum inhibitory concentrations (MICs) determined by broth microdilution. Modal MICs from triplicate testing is reported for isolates from this study. NR, not reported.

### Analysis of PBP variants in invasive SDSE isolates from Australia

**Fig 2:**
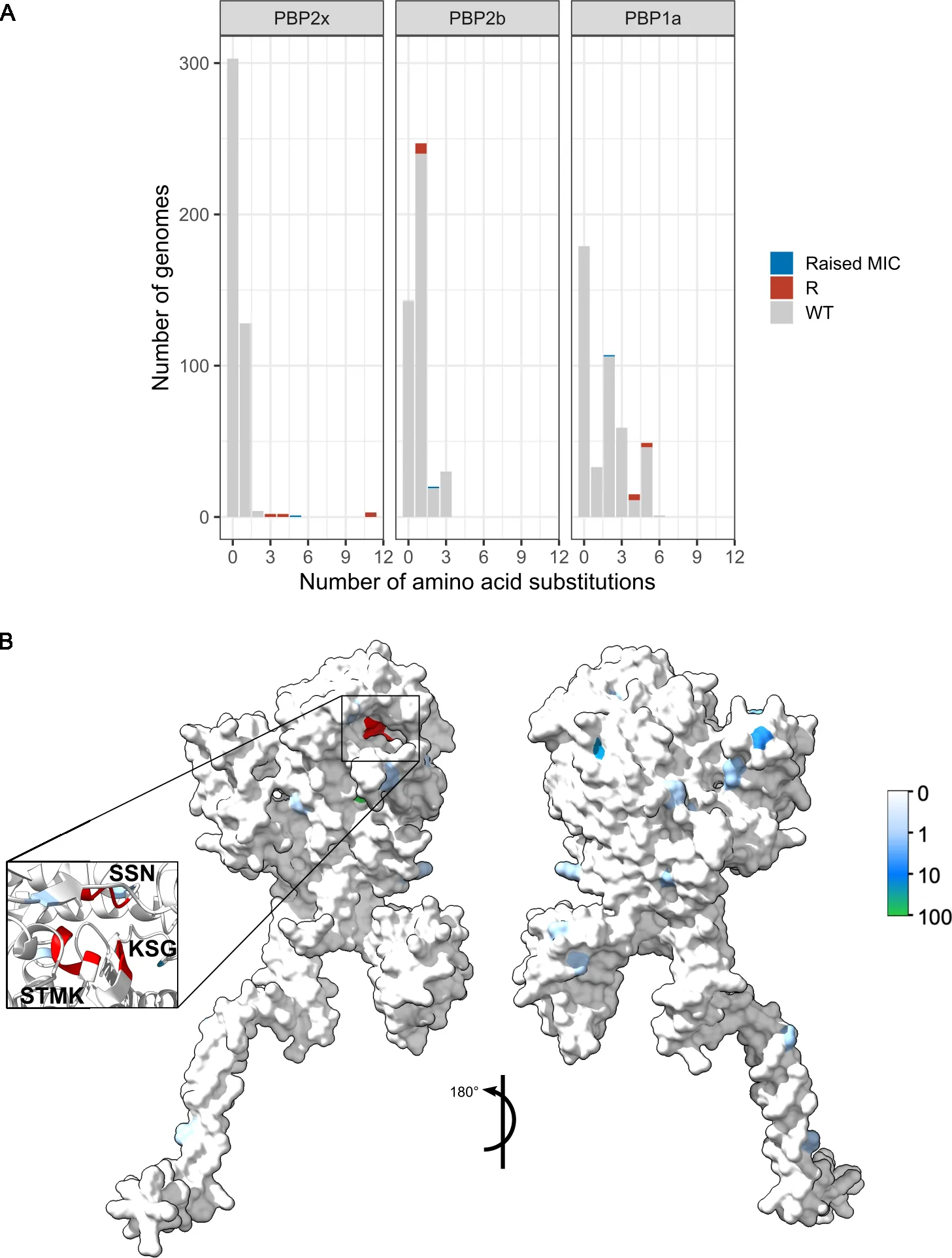
Frequency of amino acid substitutions in *Streptococcus dysgalactiae* subsp. *equisimilis* (SDSE) penicillin-binding proteins. (**A**) Frequency of amino acid substitutions in invasive SDSE cases in Australia (*n* = 436), with seven penicillin-resistant cases from this and previously published studies added for context. Gene truncations in PBP2b were not included in the count. A single invasive isolate demonstrated a four-fold increase in penicillin MIC. (**B**) Amino acid mutations of invasive isolates mapped against the predicted structure of PBP2x. The frequency of isolates with each mutation is colored from blue to green on a log scale. The STMK, SSN, and KSG active-site motifs are highlighted in red. Inset: zoomed-in ribbon diagram of the binding pocket.

To examine the circulating diversity of PBP variants, we examined 436 previously described invasive SDSE isolates collected in Australia from 2011 to 2023 (2). Within this collection, which has previously been shown to cover most sequenced global lineages, there were 22 unique PBP2x variants. The WT amino acid profile was present in 303 isolates (69%). The median number of PBP2x mutations per isolate was 0 (range 0–5), and only a single isolate had more than two amino acid substitutions (Fig. 2; Table S1a). There were 29 PBP2b variants excluding three isolates with predicted gene truncations (Table S1a). WT PBP2b was found in 143 isolates (33%). The median number of PBP2b mutations per isolate was 1 (range 0–3). There were 29 PBP1a variants with WT encompassing 179 isolates (41%) (Table S1a). The median number of PBP1a mutations per isolate was 2 (range 0–6). PBP1a also demonstrated length variation due to a variable number of STD repeats near the C-terminus of the protein (median length 721 amino acids, range 721–730).

To examine the association between PBP variants and penicillin MICs, broth microdilution susceptibility testing was performed for 63 invasive isolates selected from the Australian invasive isolates, prioritizing unique combinations of PBP2x, PBP2b, and PBP1a variants (Table 2; Table S1b). A single invasive SDSE isolate (MMC362, sequence cluster 70, *stG*866.2, ST750) from an individual with bloodstream infection and an epidural abscess had a penicillin MIC of 0.016 mg/L, four-fold higher than the modal MIC of strains with WT PBP2x (0.004 mg/L) but below the clinical breakpoint of 0.03 mg/L. The isolate had five PBP2x mutations (Table 2; Fig. S1). A373V has previously been identified in penicillin-resistant SDSE isolates (8). There were no comparator isolates from the same global genomic sequence cluster. However, isolate RDH527 (sequence cluster 13, *stG*4831.0, ST75) with WT PBP2x was matched for PBP2b (T41A, K72N) and PBP1a (T708A, D724E) variants and demonstrated a penicillin MIC of 0.004 mg/L and amoxicillin MIC of 0.008 mg/L. Two isolates had a M342I mutation in the STMK active-site motif but did not have a detectable increase in penicillin or amoxicillin MIC.

**TABLE 2:** Penicillin-binding protein 2x variants from invasive *Streptococcus dysgalactiae* subsp. *equisimilis* isolates in Australia (n = 436) with penicillin and amoxicillin minimum inhibitory concentrations (MICs)^*a*^

PBP2x variant	Strains tested	Strains with variant	Penicillin MIC (range, mg/L)	Amoxicillin MIC (range, mg/L)
WT	24	303	0.004 (0.002-0.008)	0.016 (0.004-0.016)
A397S	1	1	0.008	0.016
A397T, Q462K	1	1	0.004	0.016
A498V	1	1	0.004	0.008
D262N	2	2	0.004 (0.004-0.004)	0.016 (0.016-0.016)
G50S, Q462K	1	1	0.004	0.016
G50V	1	1	0.008	0.016
I472V	1	1	0.008	0.016
K318R	1	1	0.008	0.008
L32F	1	1	0.004	0.016
M342I	2	2	0.008 (0.004-0.008)	0.016 (0.016-0.016)
P428L	1	1	0.004	0.016
P625S	1	1	0.004	0.008
Q168F, V542I	1	1	0.004	0.016
Q462K	9	73	0.004 (0.004-0.008)	0.016 (0.008-0.016)
Q462K, V661I	1	1	0.008	0.016
R727C	2	2	0.004 (0.004-0.004)	0.016 (0.008-0.016)
S723L	1	8	0.008	0.016
S732N	4	4	0.004 (0.004-0.008)	0.016 (0.016-0.016)
**T166N, A373V, Q565L, M593T, F599L**	1	1	**0.016**	**0.064**
T306A	1	1	0.004	0.008
V542I	5	28	0.004 (0.004-0.004)	0.016 (0.008-0.016)

^*a*^ MIC reported as the modal MIC for each variant. Variants with a significantly raised MIC four-fold higher than strains with wild type (WT) PBP2x are highlighted in bold.

Although the mutated PBP2x residues in MMC362 clustered around the penicillin-binding cleft, none were predicted to participate in direct interactions with bound penicillin (Fig. S1). However, MD simulations suggested they could potentially affect the loop from residue 563–573 and disrupt the formation of the H-bond between Y572 and E382 (Fig. S2; Movie S2). In the WT protein, this interaction is predicted to stabilize the binding pocket when penicillin is bound (Movie S1). A similar phenomenon was predicted for the three penicillin-resistant isolates from this study (Movie S3).

The PBP2b mutation A583T found in penicillin-resistant isolates in this study was detected in 11 isolates. These also included five isolates with PBP1a I234V and D724E mutations, which constituted 2/5 PBP1a mutations in penicillin-resistant isolates from this study. None were associated with a significantly raised penicillin or amoxicillin MIC.

Of the 63 isolates with susceptibility testing, 10 were recurrent cases in nine individuals with isolates from the same sequence cluster, *emm*-type, and MLST >14 days after an index episode (median 6.9 months, range 1.5–83.3). No recurrent cases developed emergent mutations in PBP2x, PBP2b, or PBP1a. One isolate reverted a PBP2x L32F mutation to WT. No isolates from recurrent cases had a significantly increased penicillin or amoxicillin MIC compared to the index case.

### Global diversity of PBPs

**Fig 3:**
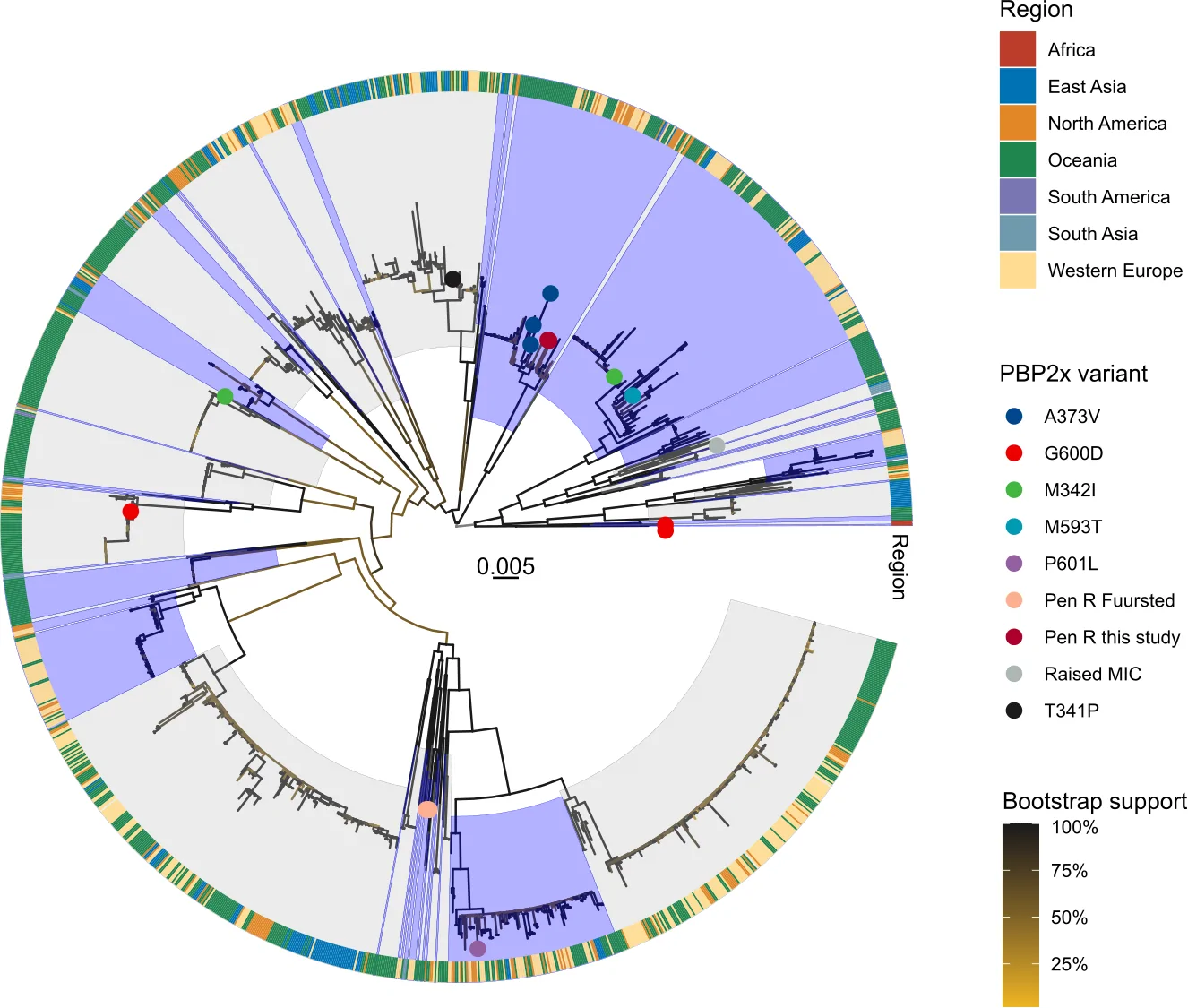
Genomic relationship of *Streptococcus dysgalactiae* subsp. *equisimilis* (SDSE) strains with PBP2x variants found previously in isolates with raised MICs or resistance to penicillin. Maximum likelihood phylogeny (*n* = 1,603) including a global database of SDSE genomes, invasive SDSE isolates from Australia, and penicillin-resistant isolates from this and a previously reported study. Distinct lineages are highlighted by alternating blue and gray shades from internal notes. Points at the tips of the tree denote four penicillin-resistant (Pen R) isolates from Fuursted et al., (8), three penicillin-resistant isolates from this study, one isolate with a four-fold increase in penicillin MIC, and presence of PBP2x variants from these isolates in the global population. The outer ring indicates the geographic region of isolation. Branches of the phylogeny are colored by ultrafast bootstrap support.

To provide global context, we combined the invasive Australian SDSE and global SDSE databases, finding 67 PBP2x, 79 PBP2b, and 66 PBP1a variants (Table S2). In addition to the two Australian isolates with the M342I mutation, only nine global genomes had a PBP2x mutation matching those in penicillin-resistant isolates or those with significantly raised MICs (three with G600D, three with A373V, one with P601L, one with T341P, and one with M593T), and none had more than one of these mutations (Fig. 3). These mutations were found in divergent sequence clusters, consistent with independent mutational events. The only isolates with more than two amino acid substitutions in PBP2x were those eight characterized above with raised MIC or resistance to penicillin (Fig. S3). There was a single genome with a T341P mutation in the STMK active-site motif accompanied by an A397S mutation. There were no other genomes with mutations in the STMK, SSN, or KSG active-site motifs.

## DISCUSSION

This study, to the best of our knowledge, is only the second report of genomically characterized penicillin-resistant SDSE causing human disease. By systematically characterizing PBP variants in a large collection of disease-causing isolates, we found an association between phenotypic penicillin and amoxicillin MICs and PBP variants. Isolates with raised MICs or resistance to penicillin had multiple PBP mutations, particularly in PBP2x. By extending genomic investigations to global SDSE genomes, we showed that amino acid variation in SDSE PBP2x was limited, and there was no evidence of expansion of lineages carrying mutations found in isolates with raised penicillin MICs.

The emergence of penicillin resistance in SDSE in all reported cases, including in this study, occurred in the setting of extensive prior or prophylactic antibiotic therapy and/or epidemiological links with other penicillin-resistant SDSE cases (8). Three out of 11 of the PBP2x mutations (G288C, M342I, and P601L) from penicillin-resistant SDSE in this study have been reported to be associated with raised penicillin MICs in *S. pyogenes*. (7, 9) However, SDSE isolates with M342I PBP2x mutations alone were not associated with raised penicillin MICs. An isogenic mutant with the M342I PBP2x mutation in *S. pyogenes* only increased the penicillin MIC 1.5-fold, which would not be readily detectable by the broth microdilution method used in this study (9).

A single invasive SDSE isolate with five PBP2x mutations was found to have a four-fold higher penicillin MIC but below clinical breakpoints. The effect of raised penicillin MICs but below the clinical breakpoint on clinical outcomes is not clear. It may lead to bacterial persistence in sub-inhibitory conditions such as with penicillin prophylaxis or difficult-to-treat deep infections without source control. The M593T mutation in this isolate has also been described in *S. pyogenes* with raised penicillin MICs (6). An invasive SDSE isolate matched for PBP2b and PBP1a variants but with WT PBP2x did not have a raised penicillin MIC supporting the association between PBP2x and penicillin susceptibility in SDSE. Reassuringly, none of the PBP2x mutations found in SDSE isolates with raised MICs or resistance to penicillin were widely dispersed in the global SDSE population.

SDSE carries five PBPs—PBP2x, PBP2b, PBP2a, PBP1a, and PBP1b—like *S. pneumoniae*. Stepwise evolutionary experiments in *S. pneumoniae* demonstrate a fitness cost when *de novo* mutations are generated in PBP2x, suggesting acquisition of multiple mutations via recombination from closely related commensal viridans streptococci are required (28). Although substantial evidence of recombination can be found in SDSE genomes, they have lower *in vitro* transformation efficacies than other streptococci (29). Further, recombination frequency decreases between more distantly related bacteria, such as between SDSE and viridans streptococci, which may naturally carry PBPs associated with penicillin resistance (30). These and other yet undefined PBP functional constraints may underlie the observation that penicillin resistance in SDSE is very rare.

This study has some limitations. While we attempted to match the invasive SDSE isolate with and without PBP variants, isogenic mutants and/or measurements of beta-lactam-PBP affinities (e.g., using fluorescently labeled penicillin analogs such as Bocillin) are required to demonstrate the causal effect on antimicrobial phenotype. As such, we cannot exclude modulating effects of other genes. There were also 42 PBP2x variants present in the global SDSE data set, which we did not have isolates available for testing. However, these comprised only 68/1,603 isolates.

In conclusion, we report a case of penicillin-resistant SDSE, which occurred under heavy selective pressure in the setting of prolonged prophylactic antibiotics. We suggest that WGS surveillance could be used to support detection of emergent SDSE populations with raised penicillin MICs. This may include searching for PBP2x mutations found in SDSE with raised MICs or resistance to penicillin, expanding lineages with novel mutations in PBP2x, and isolates with a high number of PBP2x mutations (e.g., >2). Importantly, SDSE surveillance may also have cross-species implications for *S. pyogenes,* where interspecies PBP2x recombination has been demonstrated (14, 31).

## Data Availability

Accessions for SDSE reads are listed in Tables S1 and S2. Sequences for the three new penicillin-resistant SDSE isolates are available in SRA BioProject PRJNA1314735. Sequences for invasive SDSE genomes collected in Australia are available in SRA BioProjects PRJNA1133199 and PRJNA857543.

## SUPPLEMENTAL MATERIAL

Supplemental figures and methods aac.00137-26-s0001.pdf Fig. S1 to S3 and supplementary methods.

Table S1 aac.00137-26-s0002.xlsx 436 invasive *Streptococcus dysgalactiae* subp. *equisimilis* isolates from Australia with PBP variants and molecular epidemiological markers.

Table S2 aac.00137-26-s0003.xlsx PBP variants in publicly available global *Streptococcus dysgalactiae* subsp. *equisimilis* genomes.

Movie S1 aac.00137-26-s0004.mp4 Molecular dynamic simulations of wild-type *Streptococcus dysgalactiae* subp. *equisimilis* PBP2x.

Movie S2 aac.00137-26-s0005.mp4 Molecular dynamic simulation of PBP2x with T166N, A373V, Q565L, M593T, and F599L mutations associated with 4-fold increase in penicillin MIC.

Movie S3 aac.00137-26-s0006.mp4 Molecular dynamic simulation of PBP2x with F282V, G288C, M342I, V344L, D353E, S473P, G521E, V573I, T594K, G600D, and P601L mutations associated with penicillin resistance.

ASM does not own the copyrights to Supplemental Material that may be linked to, or accessed through, an article. The authors have granted ASM a non-exclusive, world-wide license to publish the Supplemental Material files. Please contact the corresponding author directly for reuse.
