# Supplementary material for: Penicillin-resistant Streptococcus dysgalactiae subsp. equisimilis and the association with penicillin-binding protein mutations: a population genotypic and phenotypic study
Source: Antimicrob Agents Chemother. 2026 Aug 3;70(9):e00137-26. doi: 10.1128/aac.00137-26 (PMC13712881; doi:10.1128/aac.00137-26)
Supplement: Supplementary file 1 [file aac.00137-26-s0001.pdf]

Supplementary Methods

Molecular dynamic simulations

Penicillin topology was generated using antechamber in AMBER tools 2023 and converted into GROMACS topology using ACPYPE[1,2]. Amber ff99SB force field and SPC/E water model were applied for all the systems[3,4]. The complex was solvated in a periodic cubic box with wall being 1 nm away from the complex. The net charge of the system was neutralized by Cl^- ions. All bonds were constrained using LINCS algorithm[5]. The dynamics of the system at a timestep of 2 fs was generated using the Verlet algorithm[6]. The long-range electrostatic interactions were computed using the Particle Mesh Ewald method[7]. A Berendsen coupling with a coupling time of 0.1 ps was applied to maintain the temperature at 300K while a Parrinello–Rahman barostat with a pressure relaxation time of 2 ps was used to control the pressure at 1 atm[8,9]. The cut-off distance of 1.2 nm was applied for both van der Waals interactions and the short-range neighbour list. First, the system was minimized using 50,000 steps of the steepest descent algorithm and then equilibrated at constant volume over 100ps and followed by the ensemble at a constant pressure of 1 atm for 100 ps. A weak harmonic positional restraint of force constant of 1,000 kJ/mol/nm^2 was applied on the complex during the minimization and initial equilibration steps. Finally, production runs were carried out for 100 ns in triplicate at 300K in the NPT ensemble without the constraints of all the protein atoms. Animations were generated using VMD[10].

Supplementary Data

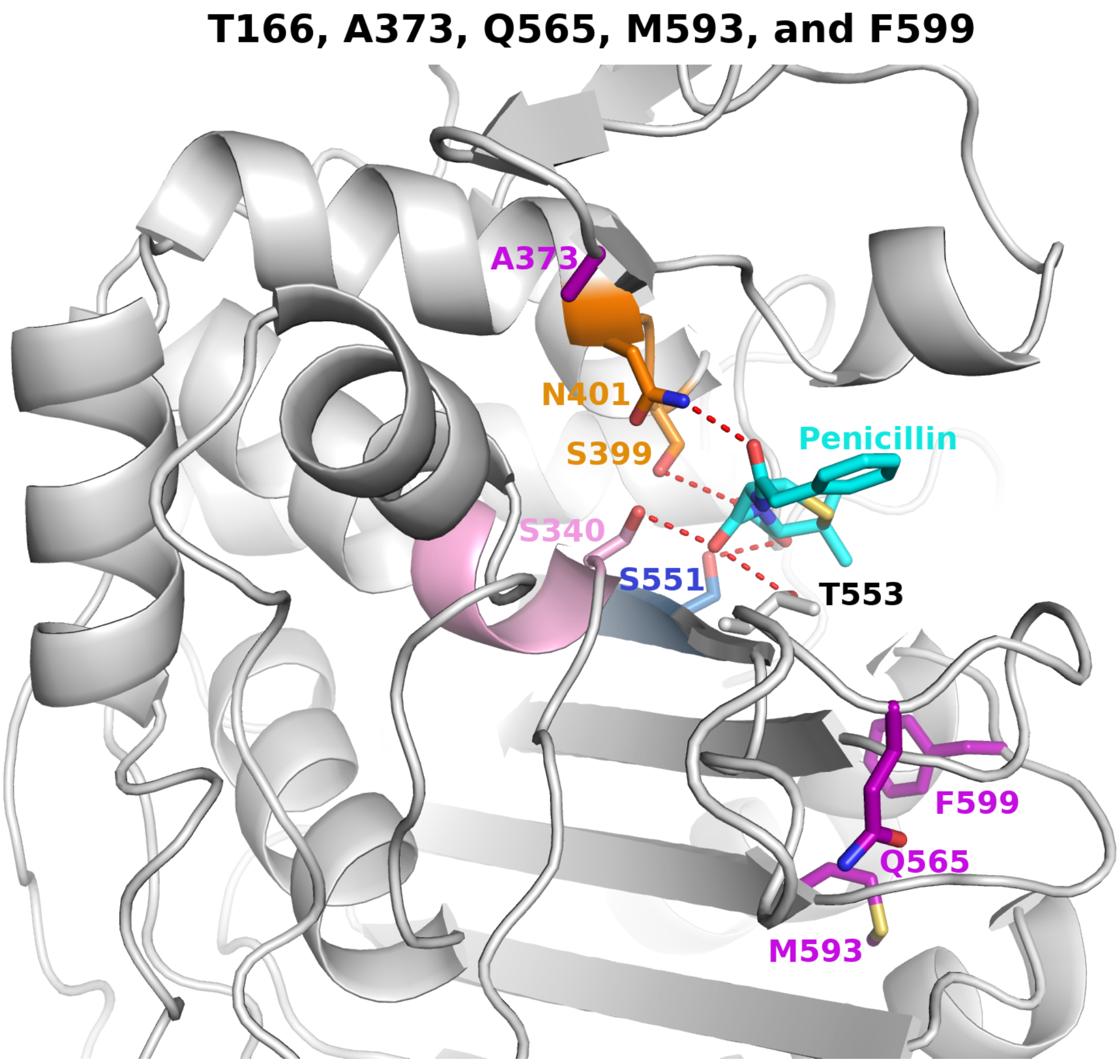

Supplementary Figure 1. PBP2x amino acid mutations present in isolate MMC362 with a 4-fold increase in penicillin MIC above strains with wildtype PBP profile but below the clinical breakpoint. Mutations are mapped against predicted structure of PBP2x (Alphafold structure AF-C5WIJ2-F1) with penicillin docked. The STMK, SSN, and KSG active site motifs are highlighted in pink, orange and blue respectively. Mutated amino acids are highlighted in purple (A373, Q565, M593, and F599). An additional mutated residue (T166) is not visible in this view. Hydrogen bond interactions with penicillin are shown in red dashed lines with nitrogen and oxygen atoms highlighted in blue and red.

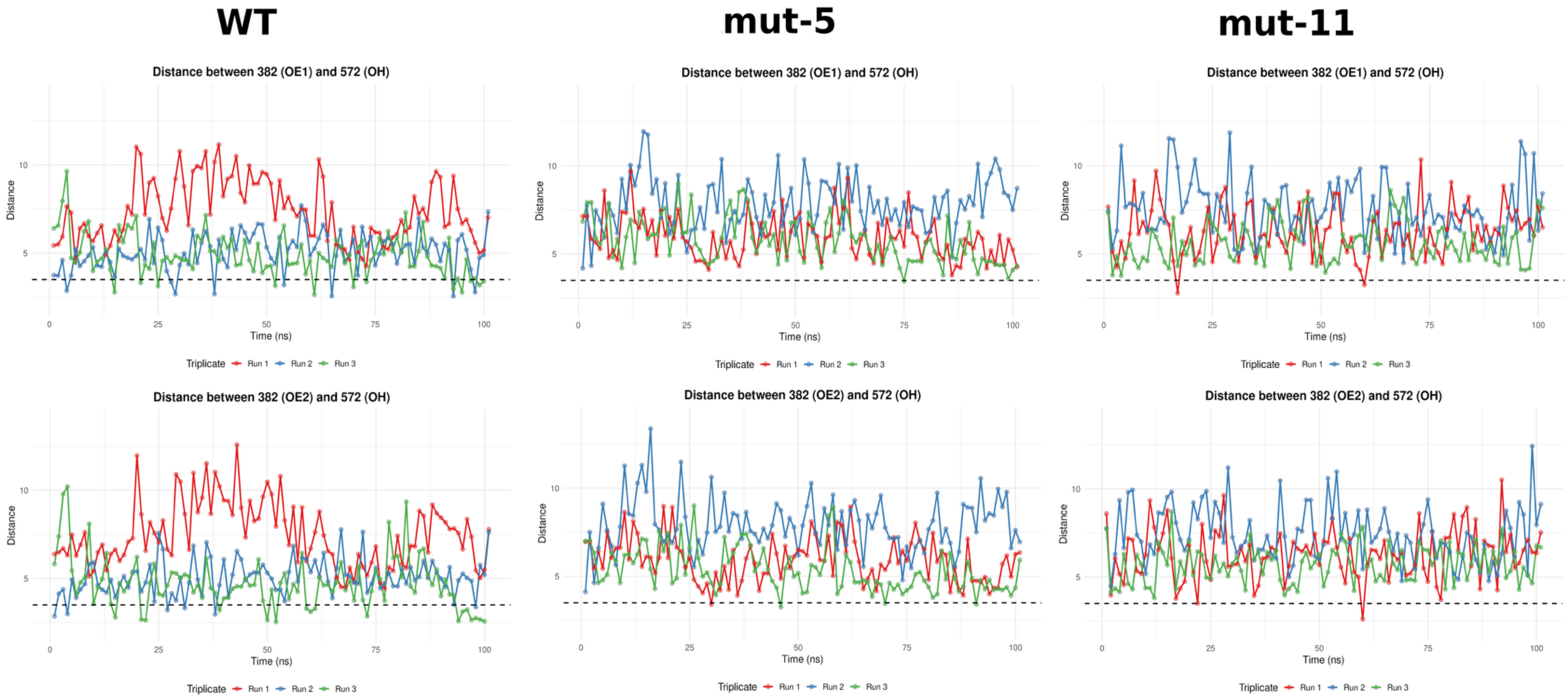

Supplementary Figure 2. Distance between side chain atoms of PBP2x residue E382 and residue Y572 for wild-type (WT), PBP2x variant with T166N, A373V, Q565L, M593T and F599L mutations associated with 4-fold increase in penicillin MIC (mut-5), and PBP2x variant with F282V, G288C, M342I, V344L, D353E, S473P, G521E, V573I, T594K, G600D and P601L mutations associated with penicillin resistance (mut-11). The cutoff for Hydrogen bond distance is shown in black dash line. Different simulations of the triplicate are shown in red, blue, and green.

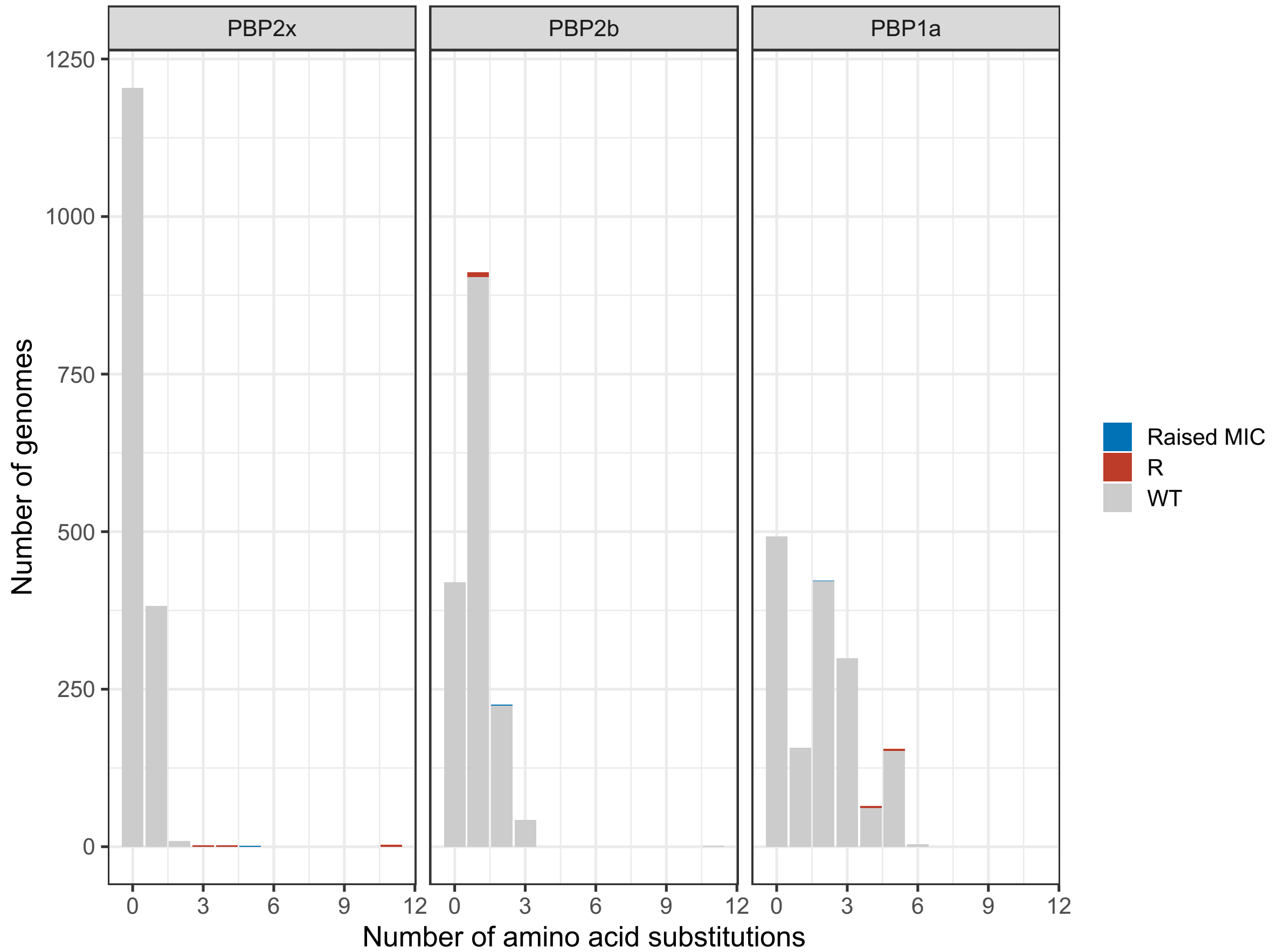

Supplementary Figure 3. Frequency of amino acid substitutions in penicillin binding proteins in global *Streptococcus dysgalactiae* subp. *equisimilis* isolates (n=1,603). A global database of 1,166 SDSE genomes were added to 436 genomes from invasive isolates in Australia and 7 penicillin resistant isolates. Six genomes had truncated or incompletely assembled PBP2x genes and were excluded

Supplementary Video 1. Molecular dynamic simulations of wild-type *Streptococcus dysgalactiae* subsp. *equisimilis* PBP2x. PBP2x is represented by the white ribbon and penicillin in ball and sticks with Carbon in cyan, Nitrogen in blue and Oxygen in red. The Y574 and E382 residues which are predicted to form an H-bond to stabilise penicillin in the binding pocket are shown in spheres with Carbon in cyan and Oxygen in red.

Supplementary Video 2. Molecular dynamic simulation of PBP2x with T166N, A373V, Q565L, M593T and F599L mutations associated with 4-fold increase in penicillin MIC. PBP2x is represented by the white ribbon and penicillin in ball and sticks with Carbon in cyan, Nitrogen in blue and Oxygen in red. The mutated residues are highlighted in purple. The Y574 and E382 residues which in the wild-type protein are predicted to form an H-bond to stabilise penicillin in the binding pocket are shown in spheres with Carbon in cyan and Oxygen in red.

Supplementary Video 3. Molecular dynamic simulation of PBP2x with F282V, G288C, M342I, V344L, D353E, S473P, G521E, V573I, T594K, G600D and P601L mutations associated with penicillin resistance. PBP2x is represented by the white ribbon and penicillin in ball and sticks with Carbon in cyan, Nitrogen in blue and Oxygen in red. The mutated residues are highlighted in green. The Y574 and E382 residues which in the wild-type protein are predicted to form an H-bond to stabilise penicillin in the binding pocket are shown in spheres with Carbon in cyan and Oxygen in red.

Supplementary references

1. Case DA, Aktulga HM, Belfon K, et al. AmberTools. *J Chem Inf Model* 2023; **63**(20): 6183–91.

2. Sousa da Silva AW, Vranken WF. ACPYPE - AnteChamber PYthon Parser interfacE. *BMC Res Notes* 2012; **5**: 367.

3. Hornak V, Abel R, Okur A, Strockbine B, Roitberg A, Simmerling C. Comparison of multiple Amber force fields and development of improved protein backbone parameters. *Proteins* 2006; **65**(3): 712–25.

4. Berendsen HJC, Grigera JR, Straatsma TP. The missing term in effective pair potentials. *The Journal of Physical Chemistry* 1987; **91**(24): 6269–71.

5. Hess B, Bekker H, Berendsen HJC, Fraaije JGEM. LINCS: A linear constraint solver for molecular simulations. *J Comput Chem* 1997; **18**(12): 1463–72.

6. Grubmüller H, Heller H, Windemuth A, Schulten K. Generalized Verlet algorithm for efficient molecular dynamics simulations with long-range interactions. *Mol Simul* 1991; **6**(1-3): 121–42.

7. Darden T, York D, Pedersen L. Particle mesh Ewald: An N·log(N) method for Ewald sums in large systems. *The Journal of Chemical Physics* 1993; **98**(12): 10089–92.

8. Berendsen HJC, Postma JPM, van Gunsteren WF, DiNola A, Haak JR. Molecular dynamics with coupling to an external bath. *The Journal of Chemical Physics* 1984; **81**(8): 3684–90.

9. Mori K, Matsumoto N, Nomoto S, Tsuruta K. Computational and experimental analyses of detachment force at the interface between carbon fibers and epoxy resin. *Open Journal of Composite Materials* 2017; **7**: 179–84.

10. Humphrey W, Dalke A, Schulten K. VMD: visual molecular dynamics. *J Mol Graph* 1996; **14**(1): 33–8, 27–8.
